# Feasibility and utility of frailty assessment in the over 80 s on critical care

**DOI:** 10.1186/cc9919

**Published:** 2011-03-11

**Authors:** B Charles, R Porter, D Bryden

**Affiliations:** 1 Sheffield Teaching Hospitals NHS Trust, Sheffield, UK

## Introduction

A recent UK-wide audit in perioperative care of the over 80 s recommended the use of frailty assessment as an independent marker of risk in older people [1]. Our critical care unit (CCU) has a fully integrated patient data management system (Metavision^®^) incorporating notes, patient data and laboratory results. We wished to determine the feasibility and utility of performing frailty assessments using our existing data collection tools on all patients over 80 years old.

## Methods

Retrospective data collection identified all patients >80 years old admitted to CCU over a 22-month period to November 2010. Frailty was assessed by means of the Canadian Study of Health and Aging index, which has been validated as a simple assessment tool [2]. APACHE II scores and numerical assessments of polypharmacy were also noted.

## Results

**Figure 1:**
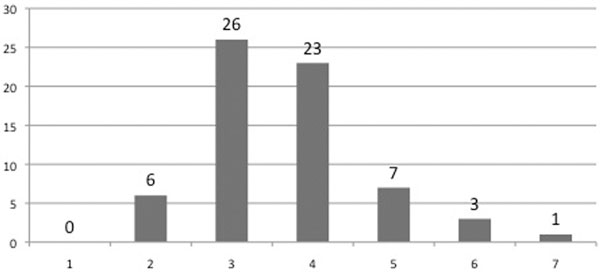
**Frequency of frailty scores**.

A total of 112 patients were identified with a median age of 83 years (80 to 92). Seventy-three per cent (*n *= 83) were discharged from critical care alive and 57% survived to leave hospital. Survival for those aged under 80 was significantly higher with 83% (*P *= 0.01) and 73% (*P *= 0.00) surviving until critical care and hospital discharge, respectively. Frailty was only able to be assessed in 66 (58.9%) of patients. Scores were as shown in Figure 1 but bore no relationship to survival. On multivariate analysis, APACHE II scores but not polypharmacy or frailty score were independent predictors of mortality.

## Conclusions

Our patients had a significantly lower unit and hospital survival than those aged under 80 and this may reflect the need for better assessment tools of frailty and co-morbidity in the critical care population. Current critical care data collection is not sufficient to adequately assess and record frailty in our unit. The National Institute for Health and Clinical Excellence will be producing a guideline for critical care in older patients and this should include a review of frailty assessment.
